# Clinical Utility of Insulin-Like Growth Factor 1 and 2; Determination by High Resolution Mass Spectrometry

**DOI:** 10.1371/journal.pone.0043457

**Published:** 2012-09-11

**Authors:** Cory Bystrom, Shijun Sheng, Ke Zhang, Michael Caulfield, Nigel J. Clarke, Richard Reitz

**Affiliations:** 1 Cleveland Heart Lab, Cleveland, Ohio, United States of America; 2 Thermo Scientific, San Jose, California, United States of America; 3 Quest Diagnostics – Nichols Institute, San Juan Capistrano, California, United States of America; Omaha Veterans Affairs Medical Center, United States of America

## Abstract

Measurement of insulin-like growth factor-1 (IGF-I) has utility for the diagnosis and management of growth disorders, but inter-assay comparison of results has been complicated by a multitude of reference standards, antibodies, detection methods, and pre-analytical preparation strategies. We developed a quantitative LC-MS method for intact IGF-I, which has advantages in throughput and complexity when compared to mass spectrometric approaches that rely on stable isotope dilution analysis of tryptic peptides. Since the method makes use of full-scan data, the assay was easily extended to provide quantitative measurement of IGF-II using the same assay protocol. The validated LC-MS assay for IGF-I and IGF-II provides accurate results across the pediatric and adult reference range and is suitable for clinical use.

## Introduction

The anabolic activity of insulin-like growth factor-1 (IGF-I) and its role in childhood growth have been extensively investigated, and quantitative analysis of IGF-I has been an essential tool in the diagnosis and treatment of human growth disorders since its measurement in serum became widely available [1]. Unfortunately, the use of IGF-I as a diagnostic tool for growth disorders is complicated by inter-assay variability that may arise from differences in antibody specificity, pre-analytical sample preparation strategies to remove binding protein interferences, non-commutability of calibration material, and dynamic range of the detection methodology [1]–[3]. From a technical perspective several key considerations are required for clinically useful determination of IGF-I: excellent precision and accuracy, within and at the extremes of normal physiological ranges; and freedom from interferences primarily considered to be associated with the presence of IGF binding proteins (IGFBPs) [4].

IGF-II is structurally related to IGF-I and binds to the IGF-I receptor, but appears to have its primary effect during gestation. Although investigated extensively as a potential biomarker in a variety of disease states, IGF-II determination is less common than IGF-I measurement and is generally used in diagnosis of non-islet cell tumor hypoglycemia [5]. While IGF-II is similarly affected by the presence of IGFBPs, the analytical demands are somewhat less given the dramatically higher circulating levels and relatively minor changes as a function of gender and age.

Recently, quantitative analysis of proteins such as IGF-I using mass spectrometry (MS) has been developed using selected reaction monitoring analysis of tryptic peptides on a triple quadrupole mass spectrometer [6]–[8]. Of particular benefit, the proteolytic digestion used in this approach would be expected to limit IGFBP interference. This strategy has been reported for thyroglobulin, a protein with known autoantibody interferences [9], [10]. Unfortunately, peptide-centric approaches are labor intensive and challenging to standardize [11]. For these reasons, we explored the potential of high-precision time-of-flight (TOF) MS for quantitative analysis of undigested IGF-I and we reported an analytical approach along with preliminary validation data [12].

The potential benefits of acquisition of full-scan MS data during quantitative analysis workflows has been described [13]. In particular, the depth of information captured in full-scan MS is appealing for cases when retrospective analysis is necessary. This is in contrast to peptide-centric approaches that use tryptic peptides as a proxy for the whole protein, in which protein structural information can be lost during enzymatic hydrolysis. Further, although selected reaction monitoring is a powerful tool for achieving selectivity, it limits the amount of structural information that can be captured in any given experiment. In the course of our method validation for IGF-I, IGF-II was identified as an additional analyte of interest due to discontinuation of a commercially available kit. Given the physicochemical similarities of IGF-I and II, we were able to develop and validate a method for IGF-II determination using substantial portions of previously collected data. This represents a clear benefit derived from the use of full-scan MS for bioanalysis.

Here we report the validation and reference range studies for the quantitative analysis of intact IGF-I and IGF-II by LC-MS. Our results demonstrate that accurate mass LC-MS approaches for proteins can offer precise quantitative measurements that meet the criteria for clinical utility.

## Materials and Methods

### Materials

HPLC-grade acetonitrile, absolute ethanol, and water were purchased from Burdick and Jackson (Morristown, NJ). High-purity formic and hydrochloric acid, bovine serum albumin, and aminoethyl benzenesulfonyl fluoride (AEBSF) were obtained from Fluka (St. Louis, MO). High-quality recombinant IGF-I was obtained from Anjinomoto Science (Raleigh, NC). Human IGF-II was obtained from R&D Systems (Minneapolis, MN). NIBSC 02/254 IGF-I reference material was obtained from NIBSC (London, UK). Rat IGF-I was obtained from Cell Sciences (Canton, MA). Recombinant IGFBP3 was obtained from Prospec-Tany (Rehovot, Israel). Surface plasmon resonance experiments demonstrating the binding of recombinant IGFBP3 to IGF-I were carried out at the Stanford Protein and Nucleic Acid facility. Charcoal-stripped human serum free of IGF-I and off-the-clot human serum were purchased from Golden West Biologicals (Temecula, CA). LiquiChek QC pools were obtained from BioRad (Hercules, CA). All protein components were fully characterized by gel electrophoresis, HPLC, high-resolution MS, and amino acid analysis prior to use (data not shown).

### Clinical sample selection

IGF-I sample selection is complicated by the biological changes that occur during pubertal development, which necessitate age- and gender-specific reference ranges. This requires large numbers of samples covering the early childhood to geriatric age range. Samples were collected from 851 pediatric subjects from ages 3.0–17.9 years, and 1240 adults between the ages of 18–85 were collected. All subjects were consented through an approved IRB. De-identified clinical samples for method development were also approved under IRB. All work represented in this manuscript requiring the use of pediatric and adult patient samples was covered under Quest IRB approval #20070882 and #20062163. Written informed consent was obtained from all adult participants or from parents or legal guardians of all pediatric participants.

In all cases, blood was collected into barrier-free serum preparation (red top) tubes, allowed to clot, and then immediately processed to obtain serum; serum was kept frozen at −80°C until analysis. Other tube types (serum with gel barrier, EDTA Plasma, Citrate Plasma, and Heparin Plasma) were also used as needed for sample-type comparison studies.

### Quantitative determination of IGF-I and IGF-II

Analysis of patient samples by LC-MS was carried out using previously described methods [12]. Briefly, IGF-I or IGF-II at 10 µg/mL was prepared in an artificial serum matrix of phosphate-buffered saline (0.01 M phosphate, 2.7 mM KCl, 137 mM NaCl, pH 7.4) containing 1 mM AEBSF and supplemented with bovine serum albumin at 45 mg/mL. Immediately before assay setup the high stock was thawed and an aliquot of the 10 µg/mL was diluted to 2000 µg/L using IGF-I/II-free stripped serum. Serial 2-fold dilutions were prepared to generate a calibration series from 2000 to 15.6 ng/mL. Internal standard was prepared from oxidized rat IGF-I in a matrix of artificial serum. For IGF-I, the m/z 1093.5209 ion from the [M+7H]+7 isotopic cluster of IGF-I was used for quantitative analysis, with the m/z 1093.3778 and m/z 1093.6641 used as qualifier ions. The m/z 1067.9385 [M+7H]+7 ion from the isotopic cluster of IGF-II was used for quantitative analysis, with the m/z 1067.7954 and m/z 1068.0817 used as qualifier ions.

For comparison purposes, subsets of patient samples were also tested using a commercial radioimmunoassay (RIA) (Esoterix, Calabasas, CA; DSL, Webster, TX), a commercial automated immunoassay (AIP) (Siemens, Tarrytown, NY), and an electrochemiluminescent immunoassay (ECL) (Meso Scale Discovery, Gaithersburg, MD).

### Determination of Reference Ranges

The dynamic and continuous change in IGF-I levels as a function of age and sex requires mathematical modeling to convert serum levels to standard deviation scores that can be used to direct diagnosis and care [14]. The IGF-I data were processed by finding the transformation for which the calculated values were closest to Gaussian distribution [14], [15]. We found that using a power of 0.4 for the entire data set minimized variance and provided the best match to a Gaussian distribution. A polynomial regression model was then fit to the transformed data using the following equation:
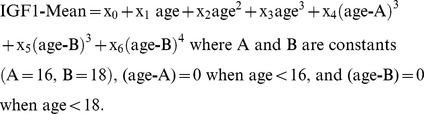
Standard deviation of the residuals about this regression model was calculated (SD = 1.379). The reference ranges were calculated from the polynomial data as (f (age)±1.96 SD)^2.5^.

IGF-II reference ranges were determined as 95% confidence intervals for the pediatric and adult populations.

## Results and Discussion

### Assay Performance and Method Comparison

**Figure 1:**
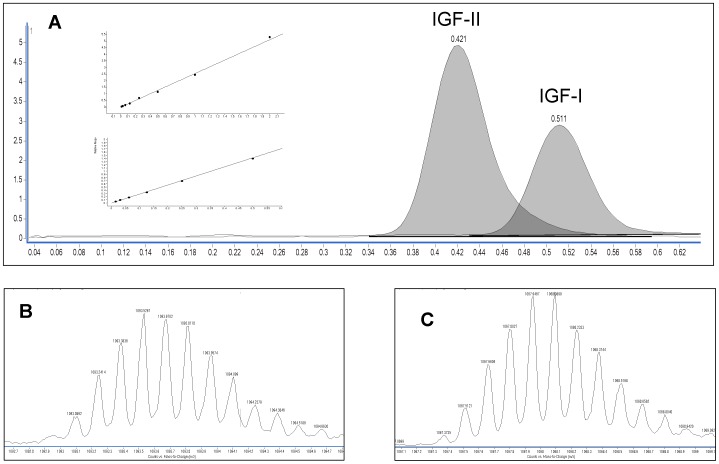
Example extracted ion chromatograms (smoothed) for IGF-I (∼250 µg/L) and IGF-II (460 µg/L) from a patient sample and inset calibration curves for both analytes (A). Representative unsmoothed MS spectra from the extracted ion chromatograms for IGF-I (B) and IGF-II (C).

Assay validation was completed to CLIA88 standards. Performance statistics for determination of both IGF-I and IGF-II from human serum are found in Tables 1 and 2 and the Supplementary data section (text S1). An example extracted ion chromatogram and representative spectrum for calibrators and patient sample are shown in Figure 1. Using Deming and linear regression and Bland-Altman plots, we compared the LC-MS method to a commercial proprietary RIA for IGF-I and IGF-II, an automated immunoassay platform (AIP) for IGF-I, and a commercial electrochemiluminescent (ECL) assay for IGF-I. The agreement between the LC-MS method and the RIA was good, but agreement with the other platforms was less favorable (Table 1). Bland-Altman plots for IGF-I immunoassay methods other than the proprietary RIA, which includes an extraction recovery adjustment, suggested non-linear behavior similar to that described by Krebs et al [16] (figure S1).

**Table 1:** Performance characteristics of high resolution mass spectrometry for determination of IGF-I and IGF-II in human serum.

Sensitivity and range (ng/mL)	IGF-I*	IGF-II
LOD	4	8
LOQ	15	30
ULOQ	2000	2000
**Target value (ng/mL), inter-assay precision (%CV), % recovery**		
BioRad low QC	100, 5.0, 104	200, 6.1, 102
BioRad medium QC	400, 5.2, 103	500, 3.2, 99
BioRad high QC	700, 3.5, 103	1200, 5.3, 99
**Established value (ng/mL), inter-assay precision (%CV)**		
Low patient pool	55, 3.3	40, 6.5
Medium patient pool	250, 3.1	227, 3.5
High patient pool	450, 2.8	450, 2.0

LOD indicates lower limit of detection; LOQ, lower limit of quantitation; ULOQ,

upper limit of quantitation; QC, quality control; RIA, radioimmunoassay; ECL,

electrochemiluminescent assay; AIP, automated immunoassay platform.

* *These data were previously published* [*12*].

**Table 2:** Comparison of LC-MS Method with RIA, ACL, and AIP by Linear Regression.

	n	Slope	Intercept	R^2^
**IGF-I**				
RIA	102	1.03	−8.1	0.98
ECL	102	0.77	56	0.93
AIP	63	0.81	11.27	0.95
**IGF-II**				
RIA	42	1.01	−49	0.78

RIA indicates radioimmunoassay; ECL, electrochemiluminescent assay; AIP, automated immunoassay platform.

Beyond the normal QC procedures requiring measurements of known quality control materials to meet pre-determined target values, we utilized analysis of isotope ratio measurements to insure the integrity of each quantitative analysis of IGF-I and IGF-II. We have found that this approach is very sensitive for detecting potential interferences [12]. Samples with potential interferences are readily identified by ion ratios that exceed tolerances, providing the opportunity to respond appropriately. To date, no evidence of an isobaric compound interfering directly with the quantifier ion has been observed. Example full-scan spectra for samples with acceptable and unacceptable ion ratios and isotope ratio performance data for IGF-II are found in the Supplementary data (figure S2, text S2).

### Assessment of IGFBP3 Interference

Given the degree to which IGFBPs can interfere with IGF assays, we investigated the sensitivity of the LC-MS approach to interference. A set of three normal patient samples with closely matched IGF-I (250±20 µg/L) and IGF-II (480±20 µg/L) levels were supplemented with functional recombinant IGFBP3 at 2, 5, 10, and 20 mg/L. IGF-I and IGF-II recoveries were determined by LC-MS, ECL, and AIP after 1 and 24 hours of incubation. No significant change in IGF-I or IGF-II recovery was observed as a function of IGFBP3 binding protein supplementation at either time point when measured by LC-MS: recoveries ranged from 99% to 106% for all measurements (repeated measures t-test for IGF-I: p = 0.81 at 1 hour and p = 0.73 at 24 hours; repeated measures t-test for IGF-II: p = 0.67 at 1 hour and p = 0.47 at 24 hours). In contrast, the ECL and AIP methods demonstrated statistically significant alterations in IGF-I recovery at 10 and 20 mg/L supplementation levels, indicating IGFBP3 interference (n = 3, mean recovery at 10 µg/L = 82%; mean recovery at 20 µg/L = 68%; p<0.01 for each level, repeated measures t-test). In addition, 15 closely matched adult patient samples with a mean IGF-I of 180 µg/L were spiked with 5 mg/L of IGFBP3 and allowed to equilibrate for 72 hours. Mean recovery of IGF-I after supplementation was 105% (range 99%–117%). Finally, as a complement to the use of recombinant material, we pooled de-identified patient samples with low (>3 mg/L) and high (>8 mg/L) levels of IGFBP3 and determined the IGF-I and IGF-II concentration. These two pools were titrated one against another (4∶1, 2∶1, 1∶1, 1∶2, and 4∶1), and IGF-I/II levels were determined after 24 hours of incubation. Expected recovery was linear as a function of dilution (data not shown), suggesting no interference from IGFBP3.

Taken together, these data indicate that the LC-MS determination of IGF-I and IGF-II is not subject to binding protein interference.

### Sample Type and Stability

In a comparison of sample types including serum (gel barrier and barrier free), heparin plasma yielded significantly lower recoveries than serum (n = 10, 83% mean recovery, range 80–88%; p<0.001 compared to serum, pairwise t-test); this effect has been reported previously [17]–[19]. EDTA (n = 10; 95% mean recovery; p = 0.21, pairwise t-test) and citrate plasma (n = 10; 97% mean recovery; p = 0.77, pairwise t-test) were deemed acceptable. IGF-I sample stability in serum was evaluated using 20 unique patient samples stored at different temperatures. Using a criterion of >90% mean recovery, samples were stable for 1 year at −80°C, 5 days at −20°C or 4°C, and 3 days at 18°C. As expected, IGF-I levels declined in samples stored at temperatures −20°C or higher, although stability was exemplary at −80°C (no significant difference after 1 year). IGF-II stability in serum was similarly evaluated and found to be 15 days at −20°C and 7 days at 4°C and 18°C. Stability for 35 days at −80°C was determined for IGF-II, but further time points have yet been collected. Of particular interest was the observation that storage stability appears to be quite short for IGF-I at −20°C (5 days), which is in contrast to published data that indicated good stability when serum was stored at −25°C [17]

### Reference Range Comparison and Clinical Utility

**Figure 2:**
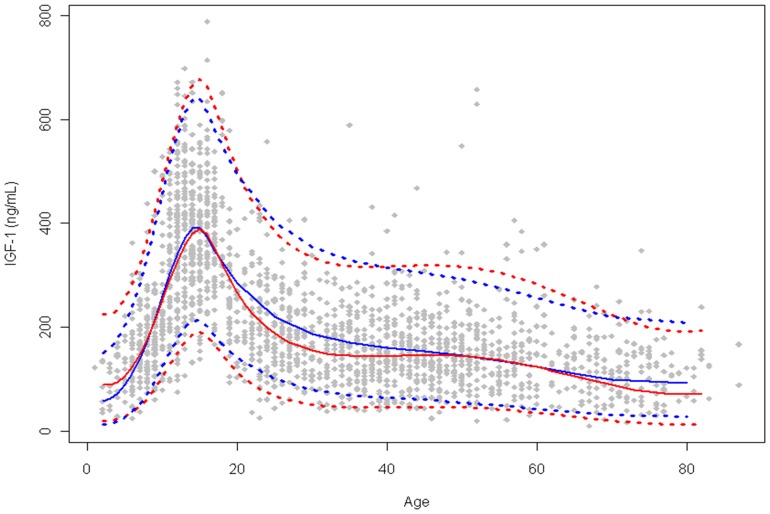
Comparison of IGF-I reference ranges determined by LC-MS (red) to immunoassay ranges reported by Brabant [**15**] (blue). Primary LC-MS data (♦), mean (solid), and 2σ reference intervals (dashed).

Determination of IGF-I reference intervals was undertaken using a set of normal pediatric and adult samples from both males and females. A large multicenter analysis of IGF-I levels published by Brabant and colleagues in 2003 is considered a key study because of its size (n = 3961) and quality of data [15]. The paper strongly suggested the need to determine assay-specific reference ranges given the difficulty of inter-assay comparison. We completed a reference range study consisting of 2091 samples. Mean and 2σ data were calculated using the approach reported by Brabant et al, and the following fit parameters were derived: x_0_, 6.75; x_1_, −0.611; x_2_, 1.37; x_3_, −0.00518; x_4_, 0.0273; x_5_, −0.0225; and x_6_, 2.90e-6; R^2^ = 0.55. The agreement of the reference range data as determined by LC-MS closely approximates the published data, which used the Nichols Institute Diagnostics Advantage automated platform (Figure 2; tabular data found in the Supplementary section, table S1).

**Figure 3:**
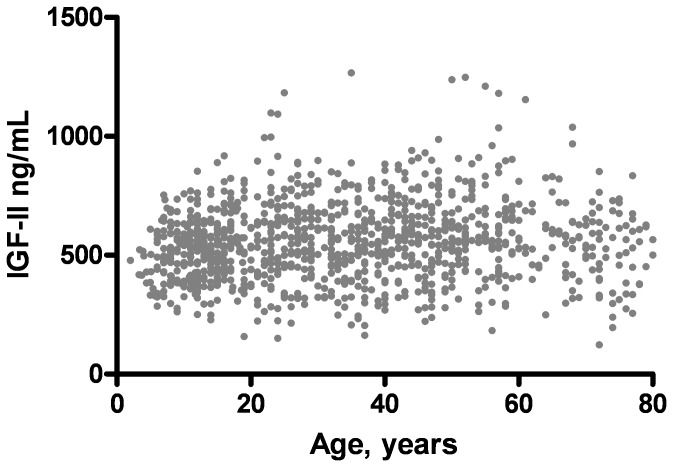
IGF-II serum concentrations as a function of age as determined by LC-MS.

In contrast to IGF-I, analysis of IGF-II levels in the same population does not reveal the dramatic age-dependent change in levels seen for IGF-I. However a statistically significant difference between pediatric and adult samples was observed, and reference ranges were established as the 95% ranges for the two populations (Figure 3, tabular data found in the Table S1).

In an assessment of clinical utility of the LC-MS methodology, 42 samples (ages 3–80) from patients diagnosed with Laron Syndrome were analyzed. Thirty-nine of the 42 samples had IGF-I levels below the LOQ of the assay, and most samples were at, or below, the LOD. The three samples with measureable levels had IGF-I values below 28 µg/L, placing each patient more than 2.5SD below the mean for their respective age group. IGF-II levels were also reduced (mean IGF-II = 140 µg/L) compared to normal samples (adult mean IGF-II = 550 µg/L), as has been reported in the literature [20], [21]

### Retrospective Analysis of Full-Scan Data

All full-scan MS data contain additional information that is essentially ignored during quantitative analysis, as only very small regions of each spectrum are interrogated during construction of extracted ion chromatograms. However, additional chromatograms can easily be generated after acquisition by the simple addition of additional masses for chromatographic feature interrogation. In the course of this work we took advantage of this capability and demonstrated the advantage that full-scan MS affords bioanalysis for both assay validation and developing a deeper understanding of analyte behavior.

During validation of our method for quantitative analysis of IGF-I, the commercial RIA used in our lab for the analysis of IGF-II was discontinued. Given the structural similarities of IGF-I and II, the question immediately arose whether the method could be modified for a validation of IGF-II. In this case, the physico-chemical behavior of IGF-I and II were so similar that a substantial portion of the IGF-I data could be reprocessed with the simple addition of an IGF-II calibrator to yield a fully validated assay with minimal additional work. While this is an exceptional case, it highlights the tremendous potential that can be realized using methods that collect full-scan data.

We were also interested in the degree to which protein modification might affect quantitative analysis. For example, methionine oxidation is facile and can be attributed to non-biological reactions that arise during sample handling and electrospray [22]. In addition, in early phases of assay development, stability studies of IGF-I spiked into serum revealed a time-dependent appearance of fragments of IGF-I with loss of two N-terminal amino acids. Assuming that this degradation was associated with serum peptidase activity, we expected that it might be observed in authentic patient samples. To investigate these possibilities, we re-processed data from 2495 patient samples, calibrators and QC pools and generated extracted ion chromatograms to examine the prevalence of IGF-I oxidation and various N- and C- terminal truncations that might arise from protein modification. Calculated extraction masses were used to perform semi-quantitative analysis (for IGF-I, oxidation, +15.9949 Da; N-1, m/z 1085.3722; N-2, m/z 1071.5076; C-1, m/z 1083.3700; C-2, m/z 1070.9369; for IGF-II N-1, m/z 1057.7904; N-2, m/z 1034.4956; C-1, m/z 1049.5039; C-2, m/z 1037.7906).

The IGF-I full-scan data analysis revealed the molecular features associated with our observation that commercial QC pools run in the LC-MS assay give distinctly lower concentrations and shorter stability than indicated in the kit insert. In particular, we observed that QC material had a high abundance of oxidized IGF-I, which is not explicitly quantified in the MS assay but may be detected by immunoassay. It is likely that this accounts for the 10–20% negative bias we have observed in QC materials run by LC-MS in comparison to labeled levels. In addition, we noted the time-dependent appearance of the N-2 proteolytic fragment of IGF-I when QC pools were stored for extended periods at 4°C rather than −80°C, also consistent with a shorter than expected shelf-life. The appearance of these modified forms of IGF-I in QC material does not affect the assay but can lead to discrepancies between labeled and experimentally determined values by LC-MS. It is also possible that immunoassays might be subject to differential detection of these forms, depending on the epitopes that the antibodies recognize.

**Figure 4:**
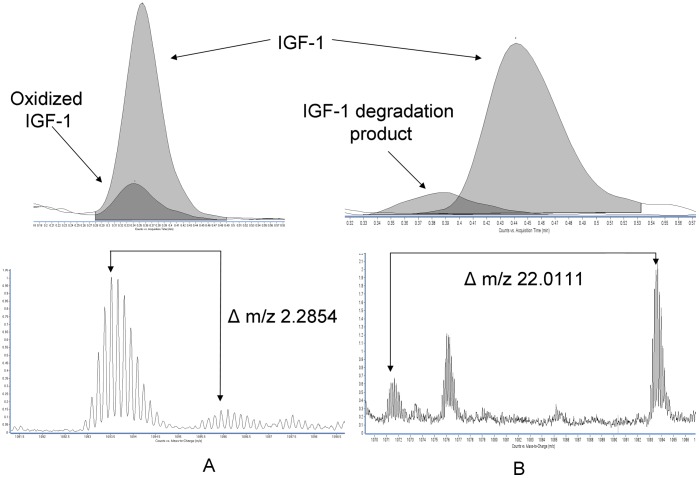
Spectra of modified IGF-I found in commercial QC pools and patient samples (5a, oxidized IGF-I in commercial QC pools, Δ m/z 2.2856 expected, Δ m/z 2.2854 observed; 5b, putative N-2 proteolytic fragment of IGF-I in a patient sample Δ m/z 22.0106 expected, Δ m/z 22.0111 observed).

In the population of samples we examined, the relative lack of modification to IGF-I was somewhat surprising. In 2495 patient samples we found only 1 instance where the abundance of oxidized IGF-I was significant enough to be considered above the limit of detection. In this case, the abundance of the oxidized form was estimated by peak area comparison to be 10% of the non-oxidized form (Figure 4a). We did find 3 patient samples where IGF-I revealed signs of N-2 proteolytic degradation but again, the abundance of these fragments was estimated to be less than 5% of the intact form (Figure 4b). In contrast, the N-1 form of IGF-II originally described by Nedelkov and colleagues [23] was observed in all patient samples. However, no other modified forms of IGF-II were detected in any significant amount.

### Conclusions

The use of high-precision MS for the determination of IGF-I and IGF-II by LC-MS analysis of the intact protein has proven to be a powerful technique. The ease and simplicity of making direct measurements on proteins without need for generating peptides via tryptic digest greatly simplifies workflows, reduces cost and complexity, while offering very good accuracy and precision. In addition, this work demonstrates that LC-MS approaches can provide sensitivities that are competitive with currently available immunoassays.

A key feature of MS approaches is that they can be carried out under conditions that can be tuned to preserve or eliminate biologically relevant interactions. In this case, the fact that the entire extraction and chromatographic separation are carried out under acid conditions, where IGF-I is liberated from its biological complex, is likely to be largely responsible for the lack of interference from IGFBP3 [3]. Although additional forms of IGFBP are known to bind IGF-I, the dissociation properties under acid conditions are general and these forms are not expected to interfere [24], [25].

Of special significance is the degree to which properly designed MS assays detect or ignore protein modifications. The structural information that is carried in mass spectral data can provide a level of detail that is not routinely available using immunological methods. In the case of IGF-I, we have taken advantage of the ability of high-precision MS to reveal molecular features that are associated with discrepancies in analytical measurements. For example, we were able to observe both oxidation and N-terminal degradation in commercial QC material, which explained differences between labeled and observed IGF-I levels and storage stability. In terms of quantitative measurement and assay design, development of this high-performance assay was facilitated by the stability and lack of modifications to human IGF-I; assay development would be more complex for proteins that demonstrate a range of modifications.

We propose that for the quantitative analysis of IGF-I and IGF-II, the combination of molecular specificity, quantitative performance, availability of recombinant reference material for IGF-I (WHO 02/254), and detailed age/sex specific reference intervals make this LC-MS methodology a candidate for immunoassay replacement.

## Supporting Information

Figure S1 **Bland-Altman Plots for IGF-I Method Comparisons.**
(PPT)

Figure S2 **Example spectra for an analyses with low background and acceptable (A) ion ratios, unacceptable (B) ion ratios due to high baseline.**
(PPT)

Text S1 **Validation and Methods Criteria.**
(DOC)

Text S2 **Supplemental isotope ratio data for IGF-II.**
(DOC)

Table S1 **Tabular IGF-1 reference ranges by year.**
(DOC)
