# Supplementary figures and images for: Clinical Utility of Insulin-Like Growth Factor 1 and 2; Determination by High Resolution Mass Spectrometry
Source: PLoS One. 2012 Sep 11;7(9):e43457. doi: 10.1371/journal.pone.0043457 (PMC3439428; doi:10.1371/journal.pone.0043457)

## Slide 1
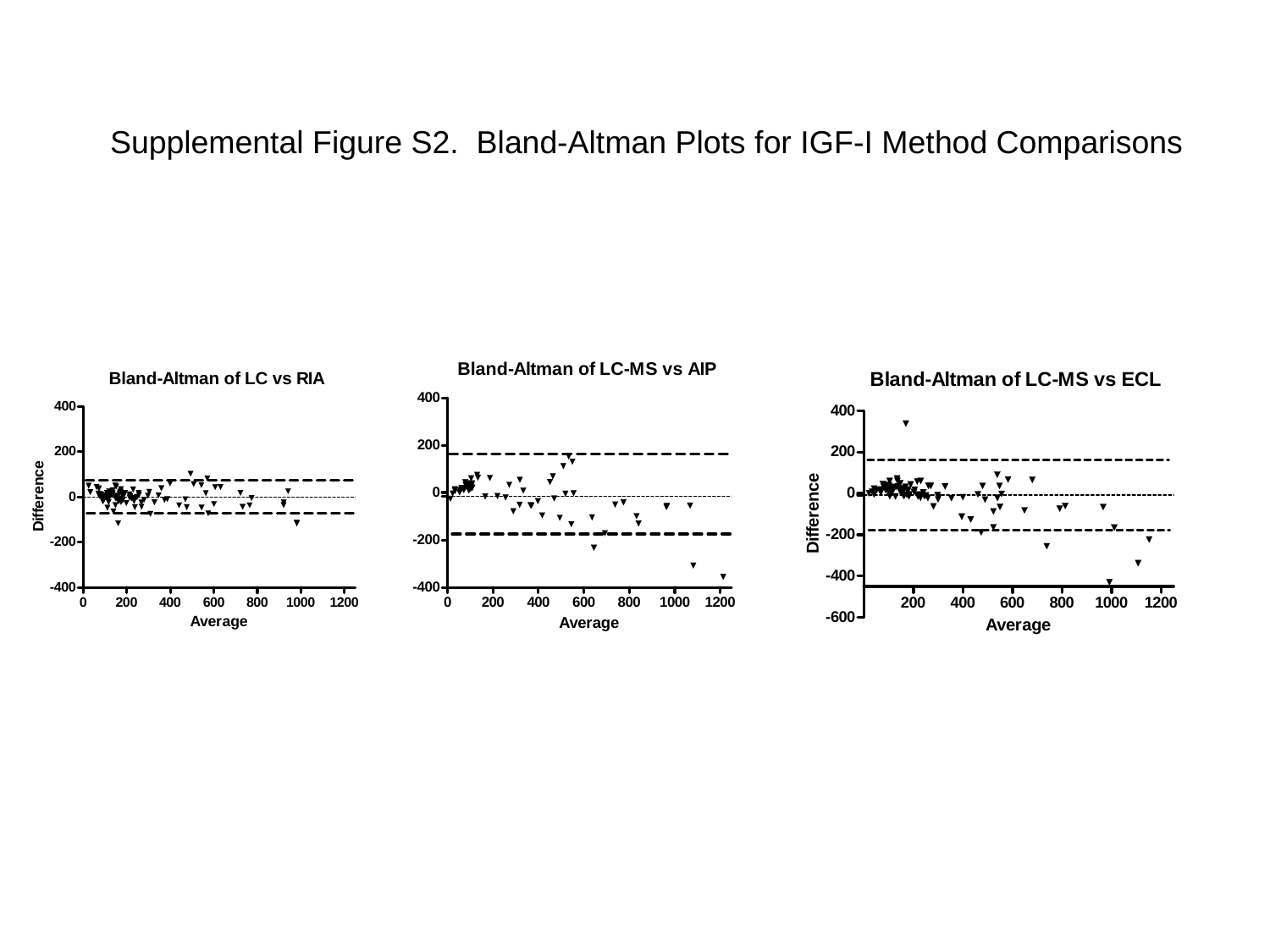

Supplemental Figure S2. Bland-Altman Plots for IGF-I Method Comparisons

Supplement: Figure S1 — Bland-Altman Plots for IGF-I Method Comparisons. (PPT) [file pone.0043457.s001.ppt]
