# Supplementary material for: Clinical Utility of Insulin-Like Growth Factor 1 and 2; Determination by High Resolution Mass Spectrometry
Source: PLoS One. 2012 Sep 11;7(9):e43457. doi: 10.1371/journal.pone.0043457 (PMC3439428; doi:10.1371/journal.pone.0043457)
Supplement: Figure S2 — Example spectra for an analyses with low background and acceptable (A) ion ratios, unacceptable (B) ion ratios due to high baseline. (PPT) [file pone.0043457.s002.ppt]

## Slide 1
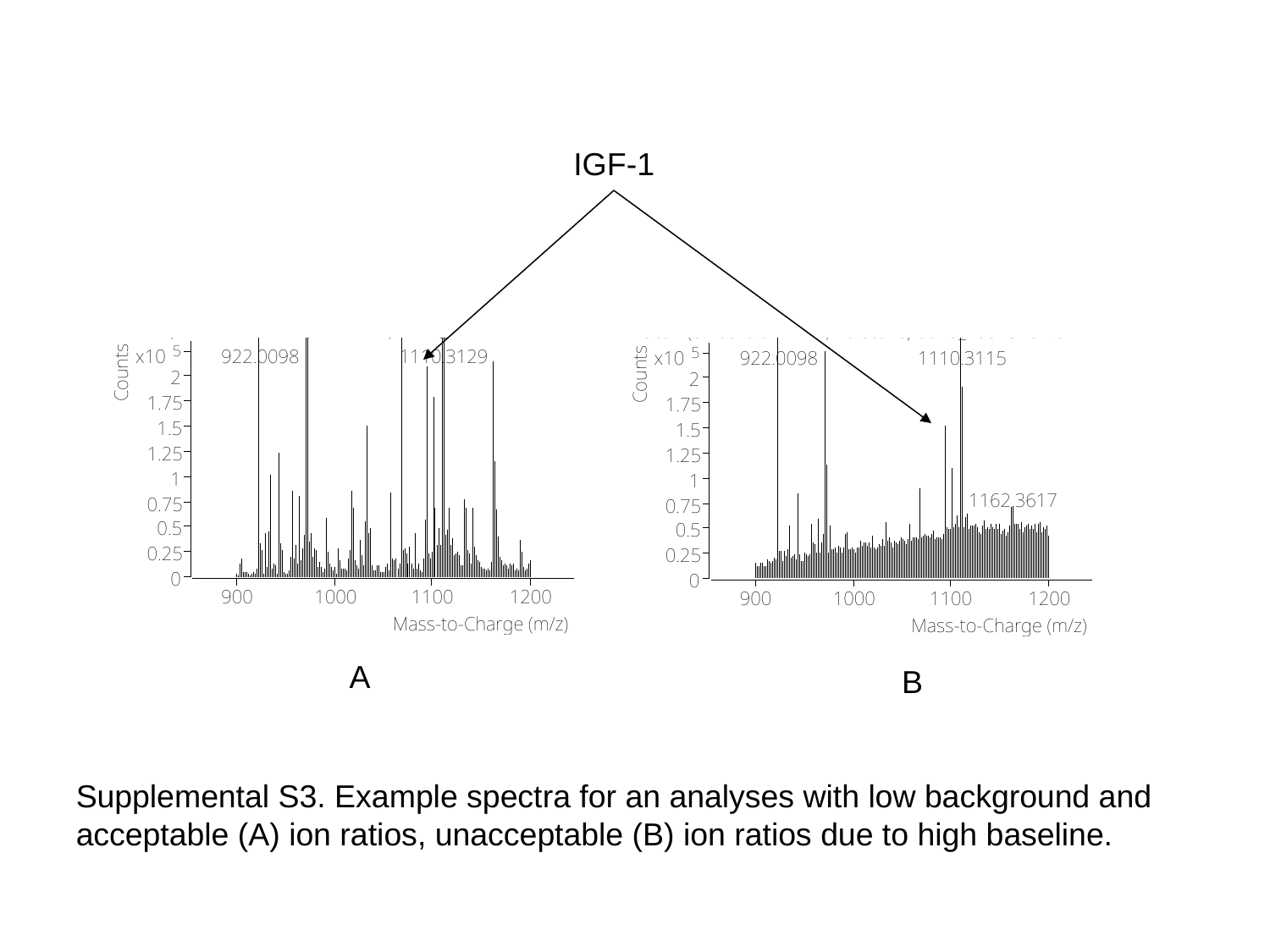

IGF-1
A
B
Supplemental S3. Example spectra for an analyses with low background and acceptable (A) ion ratios, unacceptable (B) ion ratios due to high baseline.
