# Supplementary material for: Clinical Utility of Insulin-Like Growth Factor 1 and 2; Determination by High Resolution Mass Spectrometry
Source: PLoS One. 2012 Sep 11;7(9):e43457. doi: 10.1371/journal.pone.0043457 (PMC3439428; doi:10.1371/journal.pone.0043457)
Supplement: Text S1 — Validation and Methods Criteria. (DOC) [file pone.0043457.s003.doc]

**Supplemental section S1**

**Validation methods and criteria**

Calibrator linearity was determined with charcoal stripped human serum spiked with 15.6 to 2000 ug/L recombinant IGF-I and IGF-II. Calibrator performance for each assay was expected to return back calculated values with less than 20% deviation for any individual calibrator and a correlation coefficient of >0.98 after 1/x weighted linear regression. In addition, dilution linearity was examined by determining recoveries at 2, 5 and 10x dilution of 10 patient samples with stripped serum with an expected mean recovery of >90% at each dilution. The limit of detection (LOD) was measured by running 20 replicates of stripped serum to establish the mean and standard deviation of the back calculated areas. The LOD value was set to the mean plus three standard deviations. To determine the lower limit of quantitation (LLOQ), five low pools ranging from 3.9 ng/mL to 60 ng/mL were measured five times each day over six days. The LOQ was determined as the concentration at which the inter-assay %CV of replicates was less than 20% at 95% confidence. Imprecision studies were performed using three levels of spiked, stripped serum containing 50, 100, 400, and 700 ng/mL IGF-1 or 200, 500 and 1200 ng/mL of IGF-II. Eight samples of each pool were run each day over five days. BioRad Immunocheck QC pools were also used for imprecision studies for IGF-I. Intra-assay imprecision was assessed on ten replicates at each level in a single run. Inter-assay imprecision was determined using seven replicates at each level over five days. Recovery experiments were performed using 5 serum samples spiked with four levels of IGF-I corresponding to 50, 100, 400 ng/mL and 1000 ng/mL or 62, 125, 500 and 1200 ng/mL of IGF-II and calculated by dividing the spiked amount by the expected concentration with an expected mean recovery of 100±10%.
