# Supplementary material for: Clinical Utility of Insulin-Like Growth Factor 1 and 2; Determination by High Resolution Mass Spectrometry
Source: PLoS One. 2012 Sep 11;7(9):e43457. doi: 10.1371/journal.pone.0043457 (PMC3439428; doi:10.1371/journal.pone.0043457)
Supplement: Text S2 — Supplemental isotope ratio data for IGF-II. (DOC) [file pone.0043457.s004.doc]

S4. Supplemental isotope ratio data for IGF-II

In previously published work on IGF-I, statistics for ion ratio data were collected from a set of samples and compared to theory. Subsequently a large number of patient samples were interrogated to demonstrate that isotope ratio performance was stable under high-throughput conditions.

Similar data is presented here for IGF-II. For 40 selected samples representing IGF-II concentrations from 200-700 ng/mL, and extracted ion chromatogram was generated and the averaged spectrum for the peak was calculated. Relative abundances for the nearest neighbor peaks (m/z 1067.7954 and m/z 1068.0817) were calculated to determine basic statistics for ion ratios under assay conditions. As in the case of IGF-I, abundances were in good agreement to theory (m/z 1067.7954, theoretical ratio 0.856, observed ratio 0.854; m/z 1068.0817, theoretical ratio 0.966, observed ratio 0.969).

Ion ratio performance for IGF-II was then demonstrated under assay conditions using 382 patient samples. Qualifier ion ratios were stable and retained good agreement to theory (m/z 1067.7954, theoretical ratio 0.856, observed ratio 0.858, 0.053 σ; m/z 1068.0817, theoretical ratio 0.966, observed ratio 0.974, 0.054 σ ).
