# Supplementary material for: Clinical Utility of Insulin-Like Growth Factor 1 and 2; Determination by High Resolution Mass Spectrometry
Source: PLoS One. 2012 Sep 11;7(9):e43457. doi: 10.1371/journal.pone.0043457 (PMC3439428; doi:10.1371/journal.pone.0043457)
Supplement: Table S1 — Tabular IGF-1 reference ranges by year. (DOC) [file pone.0043457.s005.doc]

S5. Tabular IGF-1 reference ranges by year

| **Age** | **Mean-1.96SD** | **Mean** | **Mean+1.96SD** |
| --- | --- | --- | --- |
| 3 | 19 | 88 | 224 |
| 4 | 22 | 94 | 234 |
| 5 | 27 | 106 | 255 |
| 6 | 35 | 124 | 285 |
| 7 | 47 | 148 | 324 |
| 8 | 63 | 178 | 371 |
| 9 | 82 | 213 | 425 |
| 10 | 104 | 252 | 482 |
| 11 | 128 | 291 | 539 |
| 12 | 151 | 328 | 593 |
| 13 | 171 | 360 | 637 |
| 14 | 185 | 381 | 667 |
| 15 | 189 | 388 | 678 |
| 16 | 183 | 379 | 664 |
| 17 | 166 | 353 | 627 |
| 18 | 146 | 320 | 581 |
| 19 | 128 | 291 | 539 |
| 20 | 112 | 266 | 503 |
| 21 | 100 | 245 | 471 |
| 22 | 90 | 227 | 445 |
| 23 | 81 | 211 | 422 |
| 24 | 74 | 199 | 402 |
| 25 | 68 | 188 | 386 |
| 26 | 63 | 179 | 371 |
| 27 | 59 | 171 | 360 |
| 28 | 55 | 165 | 350 |
| 29 | 53 | 160 | 342 |
| 30 | 51 | 155 | 335 |
| 31 | 49 | 152 | 330 |
| 32 | 47 | 149 | 325 |
| 33 | 46 | 147 | 322 |
| 34 | 46 | 146 | 320 |
| 35 | 45 | 145 | 318 |
| 36 | 45 | 144 | 317 |
| 37 | 45 | 144 | 317 |
| 38 | 45 | 144 | 317 |
| 39 | 45 | 144 | 317 |
| 40 | 45 | 144 | 317 |
| 41 | 45 | 144 | 317 |
| 42 | 45 | 145 | 318 |
| 43 | 45 | 145 | 319 |
| 44 | 45 | 145 | 319 |
| 45 | 46 | 145 | 319 |
| 46 | 46 | 145 | 319 |
| 47 | 46 | 145 | 319 |
| 48 | 45 | 145 | 319 |
| 49 | 45 | 145 | 318 |
| 50 | 45 | 144 | 317 |

IGF-1 reference ranges by year

| **Age** | **Mean-1.96SD** | **Mean** | **Mean+1.96SD** |
| --- | --- | --- | --- |
| 51 | 44 | 143 | 315 |
| 52 | 44 | 142 | 313 |
| 53 | 43 | 140 | 311 |
| 54 | 42 | 138 | 308 |
| 55 | 41 | 136 | 305 |
| 56 | 40 | 134 | 301 |
| 57 | 39 | 132 | 297 |
| 58 | 37 | 129 | 293 |
| 59 | 36 | 126 | 288 |
| 60 | 35 | 123 | 283 |
| 61 | 33 | 120 | 277 |
| 62 | 31 | 116 | 272 |
| 63 | 30 | 113 | 266 |
| 64 | 28 | 109 | 260 |
| 65 | 27 | 106 | 254 |
| 66 | 25 | 102 | 248 |
| 67 | 24 | 98 | 242 |
| 68 | 22 | 95 | 236 |
| 69 | 21 | 92 | 230 |
| 70 | 19 | 88 | 224 |
| 71 | 18 | 85 | 219 |
| 72 | 17 | 82 | 214 |
| 73 | 16 | 80 | 209 |
| 74 | 15 | 77 | 204 |
| 75 | 14 | 75 | 201 |
| 76 | 13 | 73 | 197 |
| 77 | 13 | 72 | 195 |
| 78 | 13 | 71 | 193 |
| 79 | 12 | 70 | 192 |
| 80 | 12 | 70 | 192 |
